# Reply to ‘Comment on ‘Domestic light at night and breast cancer risk: a prospective analysis of 105 000 UK women in the Generations Study”

**DOI:** 10.1038/s41416-018-0092-z

**Published:** 2018-05-17

**Authors:** Louise E Johns, Michael E Jones, Minouk J Schoemaker, Emily McFadden, Alan Ashworth, Anthony J Swerdlow

**Affiliations:** 1 Division of Genetics and Epidemiology, The Institute of Cancer Research, London, SM2 5NG UK; 2 Division of Breast Cancer Research, The Institute of Cancer Research, London, SW3 6JB UK; 3 Breast Cancer Now Research Centre at The Institute of Cancer Research, London, SW3 6JB UK; 4 Division of Molecular Pathology, The Institute of Cancer Research, London, SW7 3RP UK; 5 Present Address: Nuffield Department of Primary Care Health Sciences, University of Oxford, Oxford, OX2 6GG UK; 6 Present Address: UCSF Helen Diller Family Comprehensive Cancer Center, San Francisco, CA 94158 USA

We thank Mortazi for the comment^1^ on our recent paper published in British Journal of Cancer^2^. We indeed did not collect exposure data in this cohort, starting in 2003, to test Mortazavi and Mortazavi’s hypothesis^3^ that using smartphones, tablets and laptops in the night might affect breast cancer risk. We also did not collect information on the spectral characteristics of the light in the bedroom at night; however, in a study dependent on recalled self-reported exposure of > 100,000 women, it is difficult to imagine how one could collect such data.
